# Supplementary figures and images for: Reversible Loss of Hippocampal Function in a Mouse Model of Demyelination/Remyelination
Source: Front Cell Neurosci. 2020 Jan 22;13:588. doi: 10.3389/fncel.2019.00588 (PMC6987410; doi:10.3389/fncel.2019.00588)

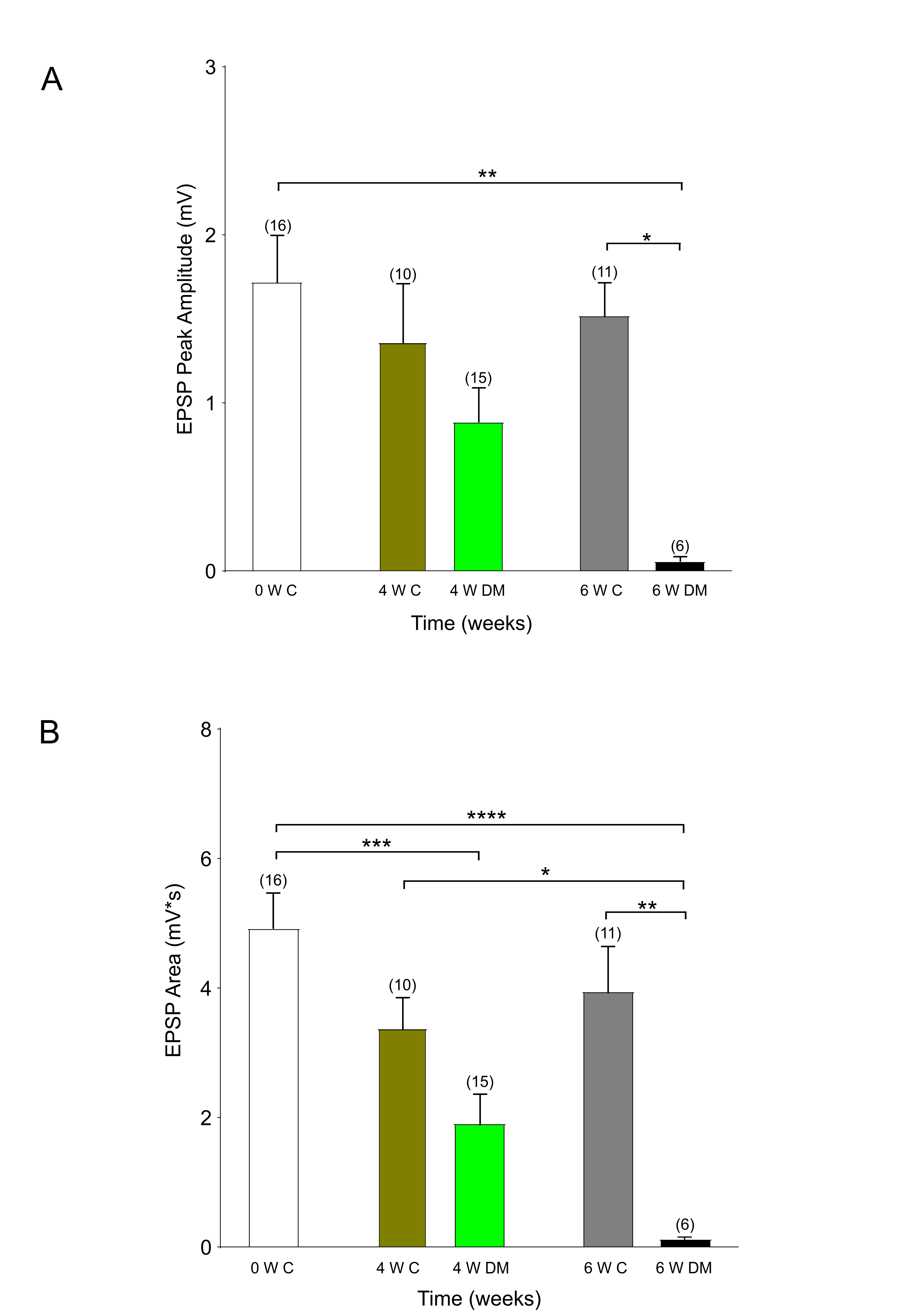

Supplement: Supplementary file 3 [file Image_1.PNG]

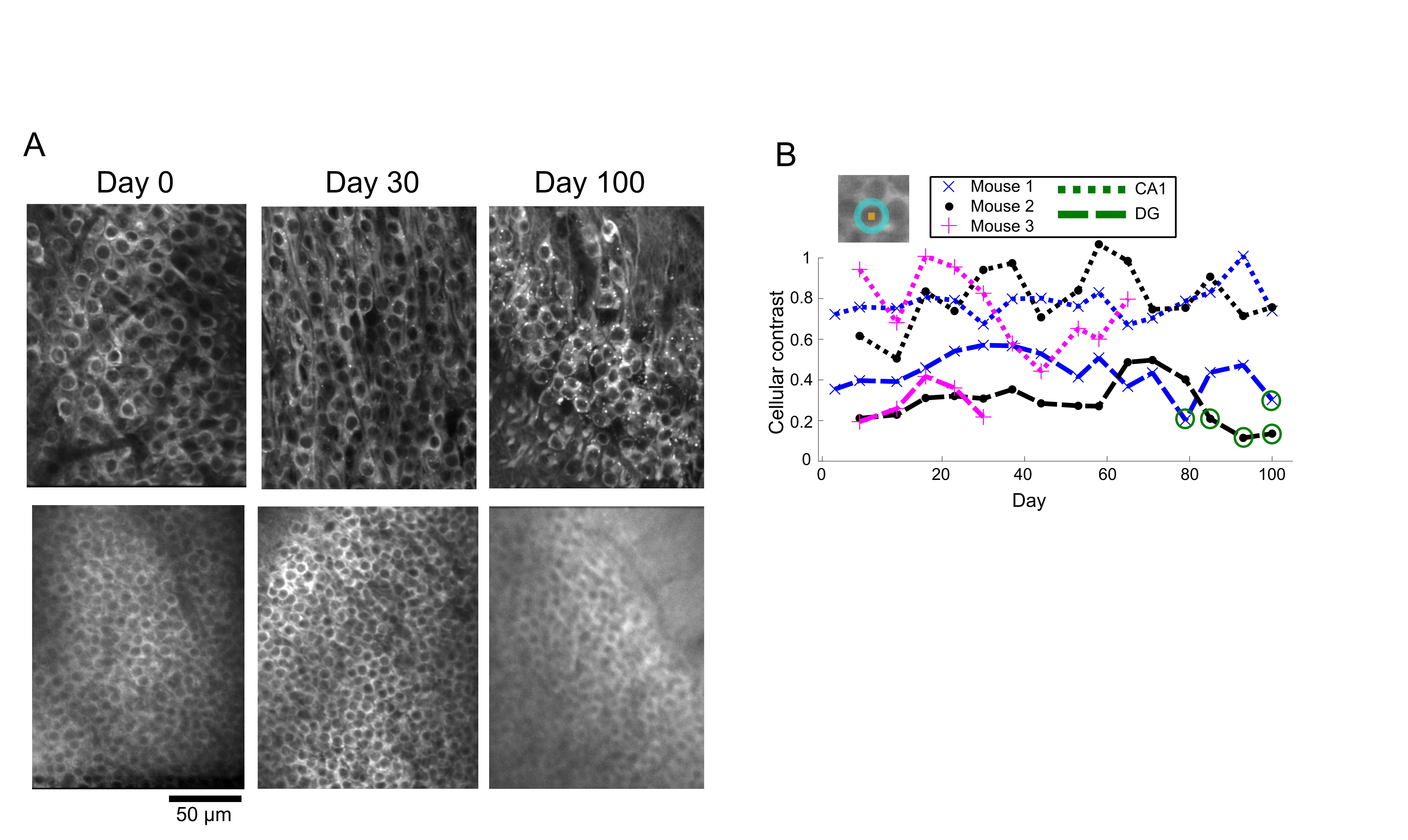

Supplement: Supplementary file 4 [file Image_2.PNG]

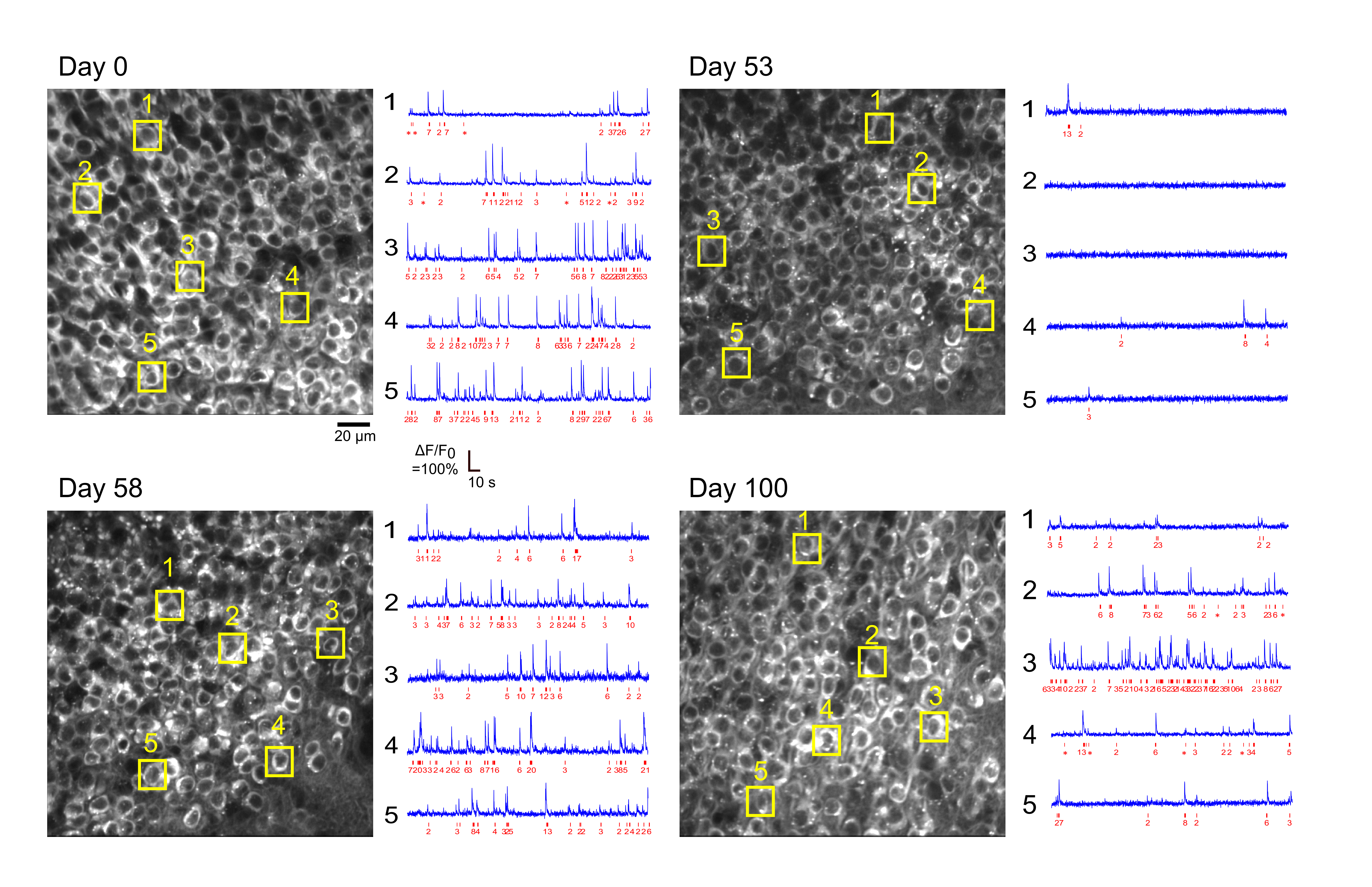

Supplement: Supplementary file 5 [file Image_3.PNG]

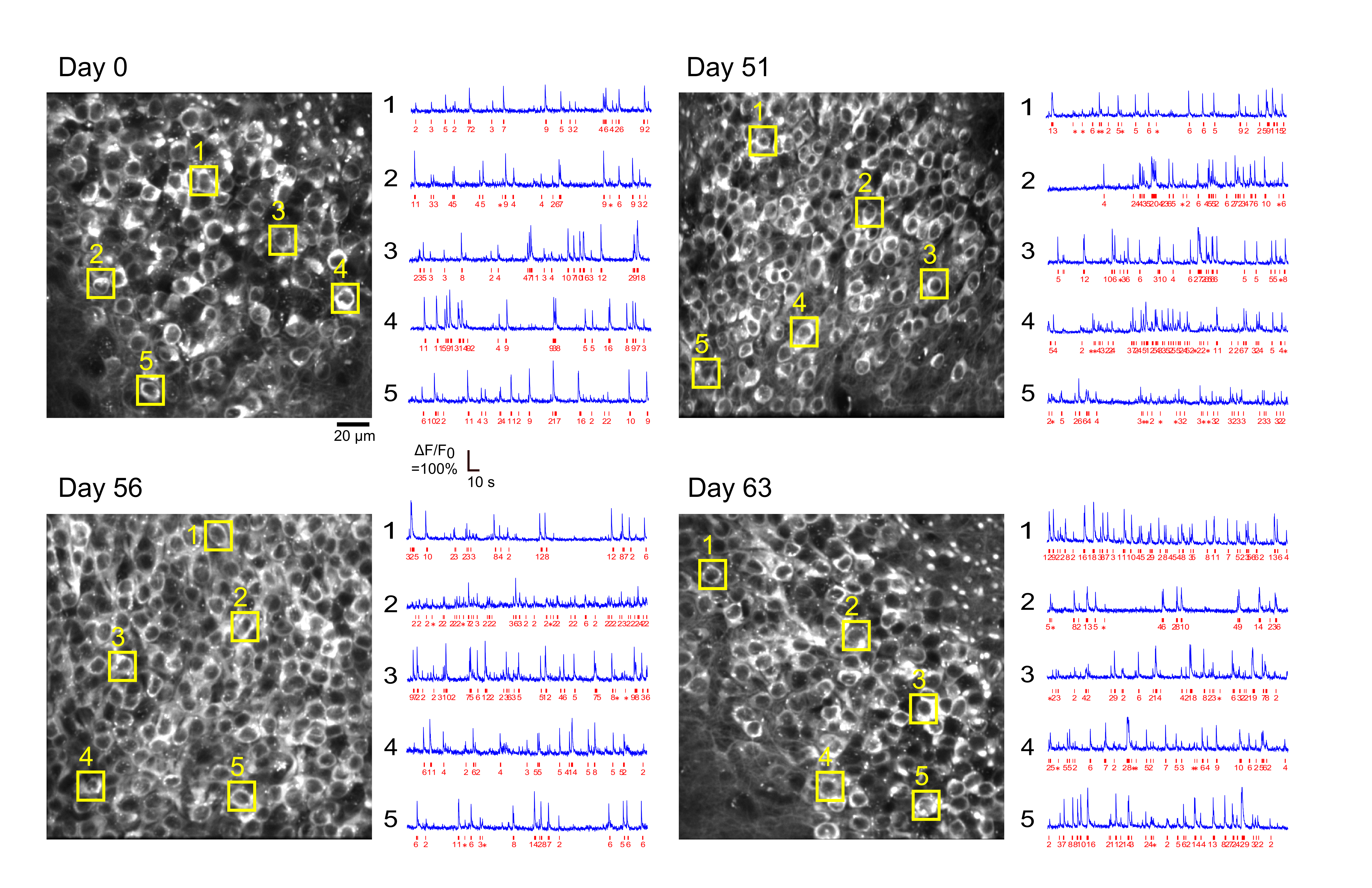

Supplement: Supplementary file 6 [file Image_4.PNG]

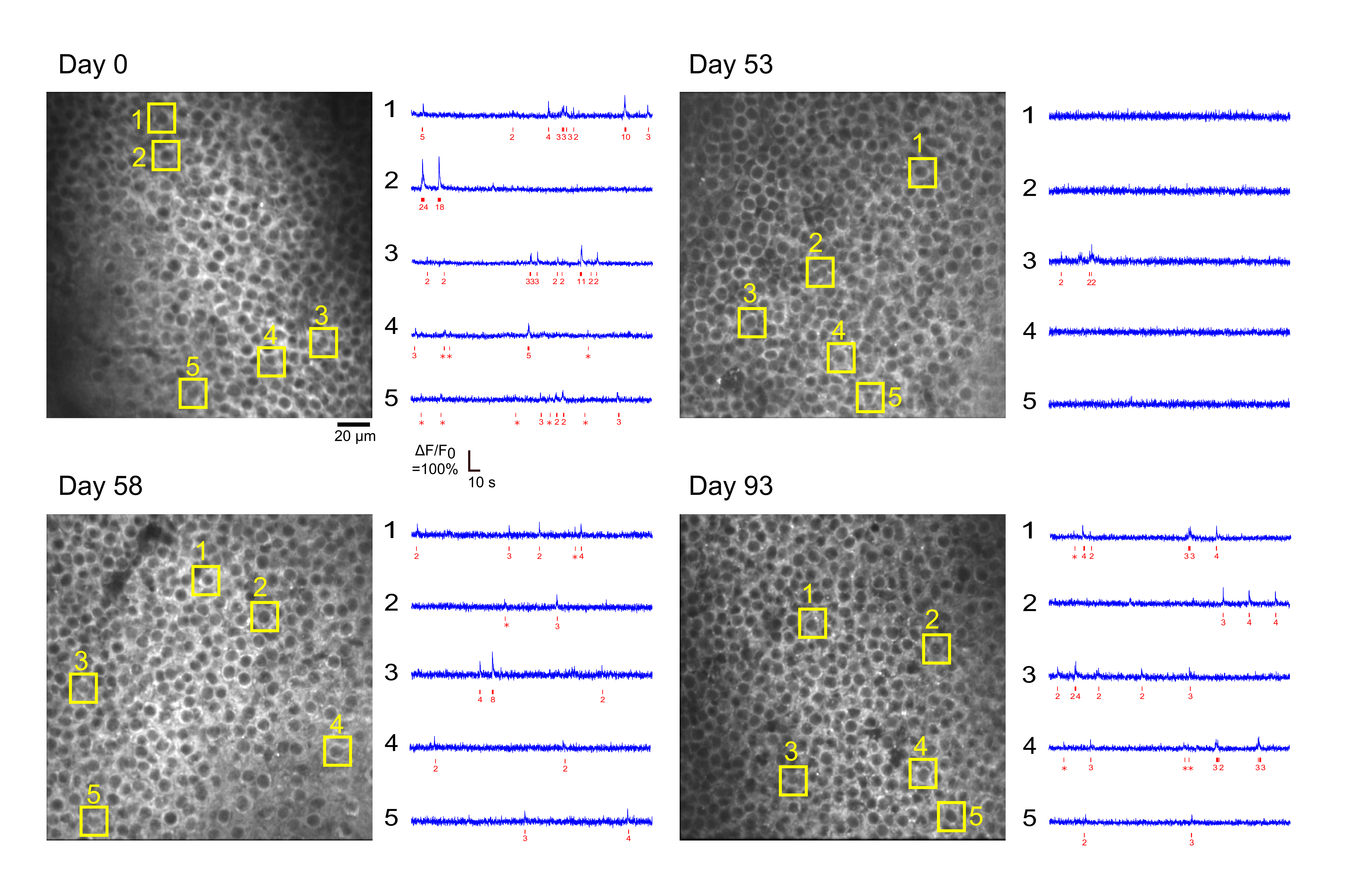

Supplement: Supplementary file 7 [file Image_5.PNG]

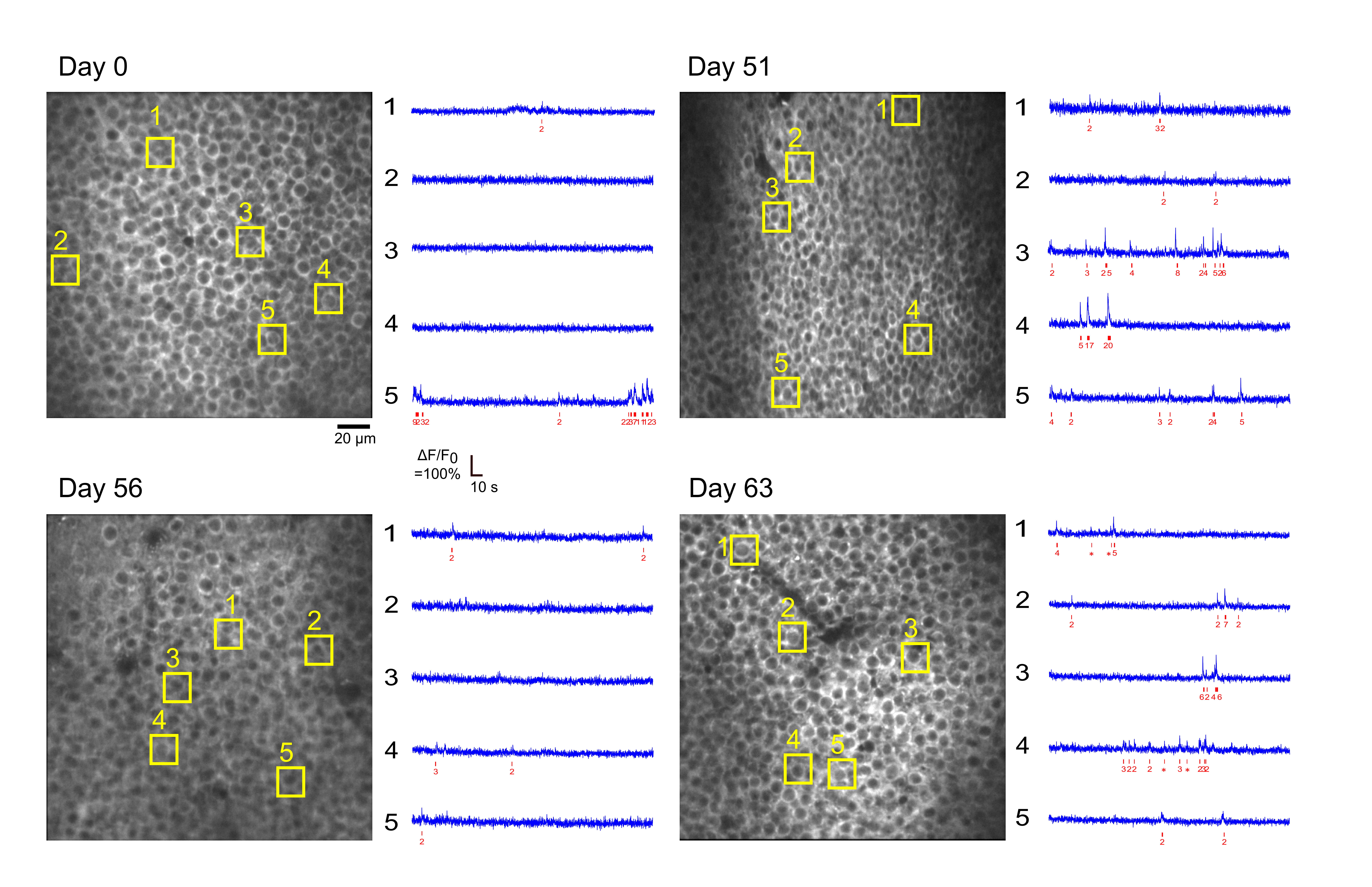

Supplement: Supplementary file 8 [file Image_6.PNG]
